# The Hospital Officers' Association

**Published:** 1902-11-22

**Authors:** 


### THE HOSPITAL OFFICERS' ASSOCIATION.

On Friday last Mr. Adrian Hope, the president of the recently-formed Hospital Officers' Association, delivered an address at the Royal Palace Hotel, Kensington, W., to a large number of members, of which the following is the substance:—

Before addressing you on the subject of my paper there are two points to which I would refer. First, you must allow me to tender you my most sincere and heart felt thanks for the honour which you have done me in electing me as the first president of your association. I am extremely proud that you should have done me this honour, and I venture to prophesy that this association will in the future afford to us all a most valuable source of education on the different knotty points of hospital management. It is often forgotten that we, who are connected with the management of hospitals, are dealing with an income and expenditure of upwards of a million pounds sterling a year in London alone, and this leads me to my second point. That is, my gratitude to you for asking me to address you to-night, which has made me feel diffident, as I find a great difficulty in addressing you, who are all experts in your various departments, upon any special point in hospital management. You all know so much better than I do the difficulties arising in the several departments more immediately within your knowledge, that I feel it would be wasting your time for me to address you upon any special details to-night. I have come here to learn from you about the improvements which can be instituted in the different departments to be found in a hospital. I have therefore decided to confine myself to-night to a forecast of the future of London hospitals in the next 10 or 20 years.

### The King's Fund.

First of all, I think that the great factor of the future will be His Majesty the King's Fund. King Edward VII. Hospital Fund for London has come to stay. It will gradually attract to itself all the large donations from those rich men who are interested in hospitals. It will more and more become the source from which we will draw our fixed income; as it becomes more powerful financially, so it will become more powerful in the matters of administration. In fact, to my mind, this fund will supply that central board for London hospitals which so many of us have thought to be needed. For myself, I welcome the prospect, though I know that to many the idea may seem distasteful at first sight, but I certainly believe that in this way, and in this way only, will improvements come. A central board for London hospitals will speak with great authority, it will compel attention from the too careless public, and it will be able to enforce improvements on hospital committees who might perhaps have shrunk from the vast cost which these improvements always entail. As regards the London hospitals themselves, I am sure that we shall see in the future some attempt at an amalgamation between some of the smaller hospitals, thus effecting a reduction of the cost of management. Looking back on the history of the last 20 years, and seeing how every hospital has struggled to achieve a convalescent home of its own in the country, I venture to suggest that the natural result of this will be an increase in the number of beds at the country or convalescent branch hospital.

### The Question of Rebuilding.

How far this increase will enable a reduction of beds in a more central situation I do not know, but of this I am certain that many hospitals will consider the question of selling their present sites and rebuilding in less expensive positions more suitable buildings with the money provided by the sale of their original sites. Each day, almost, the medical men, with whom it is our great privilege to work, demand, and rightly demand, a greater perfection in all the parts of a hospital. Our systems of ventilation, heating, lighting, and sanitary arrangements, prove more and more complicated while we strive after perfection. All this points to the necessity for the rebuilding of many of our hospitals. The convalescent hospitals in the country, I am sure, will grow and develop. Instead of having one bed in the country for every 10 beds in London, I think the tendency will be to equalise the number, if not to increase the number of beds in the country beyond the total in London. With a system of motor ambulance cars the transport of patients becomes very much easier to accomplish. These same motor cars, coming up from the country branch, could bring up the supply of milk and vegetables, and take back patients and soiled linen, for I think the country branch hospital might well become the centre for the supplying to its London parent hospital of both milk and vegetables, while the laundry work could be done there as well, effecting a considerable economy to the parent hospital.

### The Supply of Milk and Vegetables.

I recently went over a small hospital in France, where I was much struck to find that they entirely supplied themselves with vegetables grown in their own grounds, and that they derived their milk supply entirely from their own cows. It seems to me that this question of milk and vegetables is a far more important question than many think. Our supply of vegetables in London hospitals is very poor and expensive. If we grew them for ourselves we could enormously increase our use of them, and we should not have to pay the exorbitant prices that we do, besides economising in our meat bills. As for milk, I consider it to be one of the chief articles of hospital diet, and one which should be entirely under the control of the hospital from the time the milk leaves the cow-shed. We could then sterilise our milk and convey it in the sterilised bottles to the wards where it would be used. Our supply of eggs, too, could be obtained in the same manner.

### Co-operative Supply.

Then I look forward as one possible result of a central board for London hospitals to some system of co-operative supply, by which the London hospitals, instead of each buying sufficient for their own requirements in the markets, would derive the whole of their own supply from a central source, which, buying for the whole of the London hospitals, would distribute daily the provisions required, would purchase and sterilise dressings, would purchase the drugs and compounds used in each dispensary and supply all these on the cheapest and most economical lines. I am not sure whether it would be possible to throw upon such a store the work now being done in each hospital dispensary. No doubt each hospital requires a certain amount of medicines made to suit the requirements of its own staff. But I do believe that we are now on the verge of vast improvements which co-operation and centralisation will create in the management of the London hospitals. Up to now I think the tendency amongst hospitals has been to stand too much alone, to make individual efforts but not to attempt any collective efforts. We have all done our best for the separate hospitals for which we were working, but there has been little work done for the hospitals as a body. Of course we have seen the beginnings of such a movement already. We have perhaps some of us suffered under Sir Henry Burdett's mania for the collection of statistics, and yet how useful his book is at every turn. For myself I am always referring to it, and regard it as one striking proof of what may be done for London hospitals on the lines of co-operation. We are now keeping our accounts more or less in the same way, and we can check each other's expenditure and chuckle over our own economies, which we see largely written; and the extravagancies of our friends and neighbours, which assume gigantic proportions in our eyes. But still we learn from our copy of Burdett's Hospital Annual regretfully how such and such a one of our neighbours is more economical in some detail than we are, and we look into the matter and endeavour to achieve the height of our neighbour's virtue, or, at least, we find a reasonable excuse for our own want of that virtue.

### Rates and Local Interest.

Then London hospitals groan under what to me is a most vicious tax—the rates levied upon us. It seems to me that in the future these rates, while still paid by us to the local authority, will be returned to us from the equalisation of rates account, coupled no doubt with some municipal supervision. I welcome the thought of anything that will arouse local interest in our hospitals. London is now divided into all these new boroughs, and we must look forward to the time when the mayors and corporations of these new boroughs will take an interest in their local hospitals, though I fear it may be long before they will take the same interest that is displayed in the country towards the local charities. However, mainly through His Majesty the King, great interest has been awakened in the hospitals of London. Many people who never dreamt of discussing the subject are now beginning to take an interest in what to them is an entirely new point of view—their indebtedness to the London hospitals. I look forward to that interest increasing from year to year, bringing with it, no doubt, much sharper criticism of our methods and our ways of dealing with the subject, but I do not think that we have much to fear from c iticism; what we have suffered from in the past was neglect and want of interest.

Practical Suggestion.

As the first president of your association, I would venture to point out to you, its members, how greatly you may benefit each and all of us by holding regular meetings for the discussion of papers upon the many details of hospital management. What interesting details could be woven into a paper on the provisions required for the daily use of a big hospital, or the dispensary and its different branches; the uses of a works department; the possible evolution of some system by which all food consumed could be debited to each department correctly; the disentanglement of the out-patient cost from the in-patient cost; the hospital laundry and the question of its advantages and disadvantages; the making of splints and surgical appliances on the premises instead of buying these necessaries ready made; the manufacture of medicines from the raw material; the uses and advantages of disinfecting bedding, etc., on the spot. I see before this association all the possibilities of a brilliant and useful future if its members display some enthusiasm and assist each other in the desire to make perfect the work on which all alike are engaged—the scientific relief of the sick poor.

A discussion followed, in the course of which Mr. G. Q. Roberts, the house governor of the London Hospital, expressed the opinion that the individual as opposed to the collective efforts of the hospitals would prove of greater benefit to the hospitals themselves, as well as to the people who visit them.

